# Impact of Multimodal Intervention on Empathy Levels in Medical Students: A Questionnaire-Based Study

**DOI:** 10.7759/cureus.59169

**Published:** 2024-04-27

**Authors:** Mangesh Bankar, Shefali Gupta, Hansraj Kumar, Mayank Agarwal

**Affiliations:** 1 Pharmacology, All India Institute of Medical Sciences, Raebareli, IND; 2 Microbiology, All India Institute of Medical Sciences, Raebareli, IND; 3 Pharmacology, All India Institute of Medical Sciences, Deoghar, IND; 4 Physiology, All India Institute of Medical Sciences, Raebareli, IND

**Keywords:** reflection, toronto empathy questionnaire, role-play, empathy, communication

## Abstract

Background

Empathy is essential for effective doctor-patient communication. It enables doctors to understand patients' emotions and concerns, facilitating personalized care and support. Empathy can be cultivated through various methods and training programs.

Objective

The study aims to assess the effectiveness of a multimodal intervention involving interactive lectures, peer role-play, and guided reflection in enhancing empathy levels among second-year medical undergraduate students in India.

Methods

This study utilized a questionnaire-based, pre- and post-test interventional design. Seventy-nine second-year medical students were included after obtaining their informed consent. The students received the intervention through an interactive lecture on communication skills, role-play on selected case studies, and guided reflection. The empathy levels were assessed using the Toronto Empathy Questionnaire (TEQ) before and after the intervention. The Mann-Whitney U test was utilized to compare pre-test and post-test TEQ scores. A univariate analysis of variance was conducted to explore the relationship between demographic variables and post-test TEQ scores. Statistical significance was considered at p ≤ 0.05.

Results

The TEQ score improved significantly (p=0.009) after the intervention. The univariate analysis indicated that gender, style of education, and place of residence did not have a statistically significant impact on post-test scores.

Conclusion

The study demonstrates that a multimodal intervention significantly enhances the empathy level of medical students, highlighting the potential of focused interventions to reduce gender disparities in empathy levels. There were no significant differences in empathy scores based on gender, place of residence, or schooling, suggesting the intervention's benefits may apply to all medical students.

## Introduction

Effective doctor-patient communication relies heavily on empathy, a crucial aspect of interaction [1]. Empathy entails understanding and connecting with the feelings and experiences of others to foster meaningful connections. It is categorized into affective empathy and cognitive empathy. Cognitive empathy involves understanding the patient's mental state, while affective empathy involves connecting with the patient's emotional condition [2,3].

Previous research indicates that effective communication between doctors and patients significantly enhances the patient's quality of life. This communication fosters trust between both parties, making gathering detailed information about the patient's concerns easier, promoting medication adherence, and reducing the likelihood of adverse drug reactions [4]. Therefore, improving the empathy levels among medical students during undergraduate medical education is crucial for producing doctors who can provide more comprehensive and compassionate care to their patients.

According to a systematic review by Samantha et al., various educational interventions can enhance empathy among medical students [5]. The student role-play method stands out as a cost-effective intervention where students portray both doctor and patient roles. This approach enables medical students to grasp their patients' emotions and viewpoints, offering valuable insights into enhancing their interactions effectively [6]. Another study focusing on third-year medical students found that participating in a 60-minute structured reflection session led to a notable enhancement in empathy that was sustained for up to one year [7].

Unfortunately, there is a scarcity of interventional studies in India, with most previous research comprising cross-sectional surveys focused on determining baseline empathy levels among students. Additionally, several previous studies have reported low baseline empathy scores among Indian medical students compared to studies conducted elsewhere [8-10]. This highlights the need for more comprehensive multimodal interventional studies in India to address the empathy gap among Indian medical students. Lauzier et al. suggested that a multimodal teaching approach can enhance learning outcomes and reinforce behavioral changes in participants [11]. Therefore, this study was conducted to assess the impact of a multimodal intervention consisting of an interactive lecture, peer role-play, and guided reflection on the empathy levels of medical undergraduate students.

The primary objective of this study was to assess the impact of multimodal intervention on the empathy scores of medical students. The secondary objective was to explore potential differences in empathy scores based on gender, place of residence, and type of schooling.

## Materials and methods

Study design

This was a questionnaire-based, pre-test and post-test interventional study aimed at examining the impact of a multimodal intervention, comprising an interactive lecture, peer role-play, and guided self-reflection, on the empathy level of medical students.

Study setting

The study was conducted at the Department of Pharmacology, All India Institute of Medical Sciences (AIIMS), Raebareli, Uttar Pradesh, India, from March to April 2024.

Ethical consideration

The Institutional Ethics Committee, AIIMS Raebareli, approved the project (IEC no. F.3/BIOETHICS/AIIMS-RBL/APPRO/IM/2024-6/9). Before inclusion in the study, all individuals provided informed consent. Participant privacy and confidentiality were maintained throughout the study.

Sample size calculation

The sample size was determined based on the total number of medical students at AIIMS, Raebareli. The sample size was calculated using the single proportion formula [12] as follows:

n=N/(1+N*[MOE]^2^)

where N is the total number of medical students in our institute (350), MOE is the margin of error (0.1), and n is the desired sample size.

Considering 350 medical students studying at the time of participant recruitment, a sample size of 76 was calculated using the above formula. However, 84 participants were included in the study to account for potential dropouts.

Inclusion and exclusion criteria

Second-year medical students who provided their consent to participate were included in the study, and their socio-demographic information, including age, gender, type of schooling, and place of residence, was collected. Students who had received training on doctor-patient communication skills or declined to participate were excluded from the study. The students were assured that their participation in this study would be completely voluntary and that their confidentiality would be maintained. Additionally, participants were assured that their choice to participate or not would not have any adverse effect on their academic standing.

Study procedure

In the first contact session, baseline empathy scores were collected by distributing the Toronto Empathy Questionnaire (TEQ). Demographic information, including age, gender, place of residence, and type of schooling (central or state board), was also recorded.

Six independent experts with at least 10 years of experience and faculty positions in various medical colleges validated the content of the empathy teaching module. The empathy teaching module was evaluated in terms of relevance and clarity. The experts evaluated each aspect of the module on a four-point Likert scale ranging from 1 to 4. The feedback and ratings provided by the experts were carefully analyzed to ensure the validity of the empathy teaching module. Based on their evaluation, any necessary improvements were made to enhance the module's relevance and clarity. The scale-level content validity index based on the average method (S-CVI/Ave) was 0.95. Furthermore, the scale-level content validity index based on the universal agreement method (S-CVI/UA) was found to be 0.87, indicating that the module had a satisfactory level of content validity.

Subsequently, an independent facilitator conducted a brief introductory lecture on the principles of doctor-patient communication. Two YouTube videos, one on the wrong communication practices (link: https://www.youtube.com/watch?v=Hgk0_E236D4) and the other on the proper ones, were shown to the students (link: https://www.youtube.com/watch?v=OKMMIGsiCO0). After watching the videos, the students were asked to discuss and identify the differences between the two communication styles. The facilitator then facilitated a group discussion to further explore the importance of effective doctor-patient communication. Following this, student pairs were formed randomly and given a scenario where one student would play the role of a doctor and the other a patient. The pairs took turns playing the roles of the doctor and patient, focusing on active listening, non-verbal cues, and empathy. A total of 42 pairs were formed, and 20 doctor-patient communication case scenarios were presented during the role-play, covering a wide range of medical situations. After the role-play, the facilitator led the students in a guided reflection session to help them evaluate their performance and pinpoint areas for improvement. The students discussed how their communication skills influenced the doctor-patient relationship and how they could further enhance their abilities.

After the guided reflection activity, a post-test was conducted to evaluate the students' improvement in their empathy scores. The TEQ, developed by Spreng et al., consists of 16 items and employs a five-point Likert-type scale with scores of "Never = 0; Rarely = 1; Sometimes = 2; Often = 3; and Always = 4" to assess the level of empathy among college students. There are eight items with positive scores and eight items with reversed scores. This scale is divided into six subgroups, each representing different aspects of empathy-related behaviors [13]. The researchers claimed that the reliability study's Cronbach's alpha value for the TEQ was 0.85. Within the context of the criterion-related validity research, the TEQ showed a substantial positive correlation with a scale similar to it and a negative correlation with a distinct scale, which supports the TEQ's ability to measure empathy accurately. Permission to use TEQ for non-commercial research and educational purposes with proper reference to the original work was obtained from the author.

Statistical analysis

The data were entered into a Microsoft Excel spreadsheet, and later, statistical analysis was conducted using IBM SPSS Statistics for Windows, Version 26.0 (Released 2019; IBM Corp., Armonk, New York, United States). The results were expressed as a mean ± standard deviation (SD) for quantitative variables and frequency (percentages) for qualitative data. The Mann-Whitney U test determined the difference between the pre-test and post-test scores based on gender, type of schooling, and place of residence. The Kruskal-Wallis test was used to compare TEQ scores among different age groups. The difference between each TEQ subscale's pre-test and post-test scores and the total TEQ scores was evaluated using a Wilcoxon-signed rank test. The Spearman rho correlation was calculated to measure the strength of the association between empathy scores and age. Univariate analysis of variance was used to examine the relationship between variables such as gender, type of schooling, and place of residence and the post-test TEQ scores after satisfactorily testing the assumptions such as normality and homogeneity of variances. Statistical significance was considered at p ≤ 0.05.

## Results

Sociodemographic characteristics of the participants

The final analysis included data from 79 medical students, as five students were excluded due to their low response rate to the TEQ questionnaire, ensuring the reliability and precision of the results. The socio-demographic information collected for this study included age, gender, place of residence, and type of schooling (central or state board), which are detailed in Table 1.

**Table 1:** Socio-demographic characteristics of the participants.

Variable (total number of participants=79)	Level	Count	Proportion
Gender	Female	27	0.342
	Male	52	0.658
Age (years)	≤20	18	0.227
	21-22	53	0.670
	≥23	8	0.101
Schooling	Central board	57	0.722
	State board	22	0.278
Place of residence	Rural	30	0.380
	Urban	49	0.620

Participants' ages ranged from 19 to 24 years, with the highest representation observed in the 21-22 age group, comprising 67% of participants, followed by the 20-year age group (22%). The mean age of participants was 21.177 years, with a SD of 1.059. Male participants predominated, constituting 65% of the sample. Sixty-two percent (62%) of the participants were from urban areas, and 72% attended central board schools.

TEQ scores of the participants

**Figure 1:**
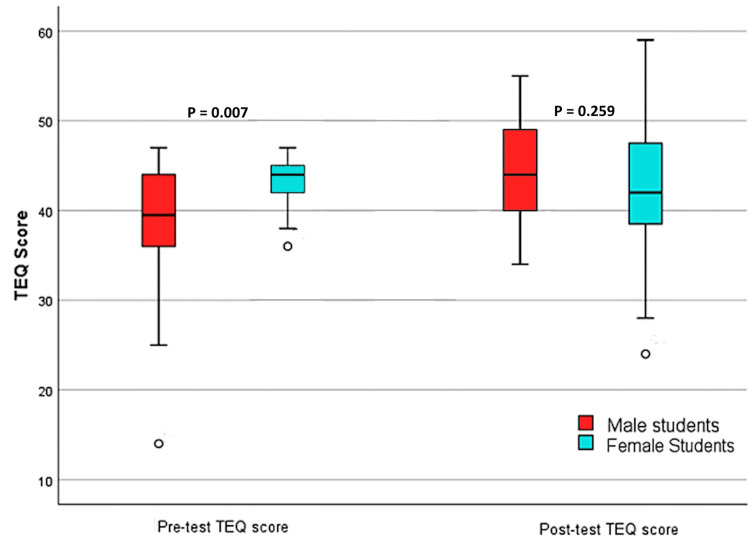
Boxplot showing the gender-wise distribution of TEQ scores of medical students. TEQ: Toronto Empathy Questionnaire.

Figure 1 illustrates the variation in participants' scores based on gender. The females had a statistically significant (p=0.007) higher baseline score than the male participants. The TEQ scores improved in both genders after the intervention. The post-test scores did not differ significantly (p=0.259) for male and female participants.

**Figure 2:**
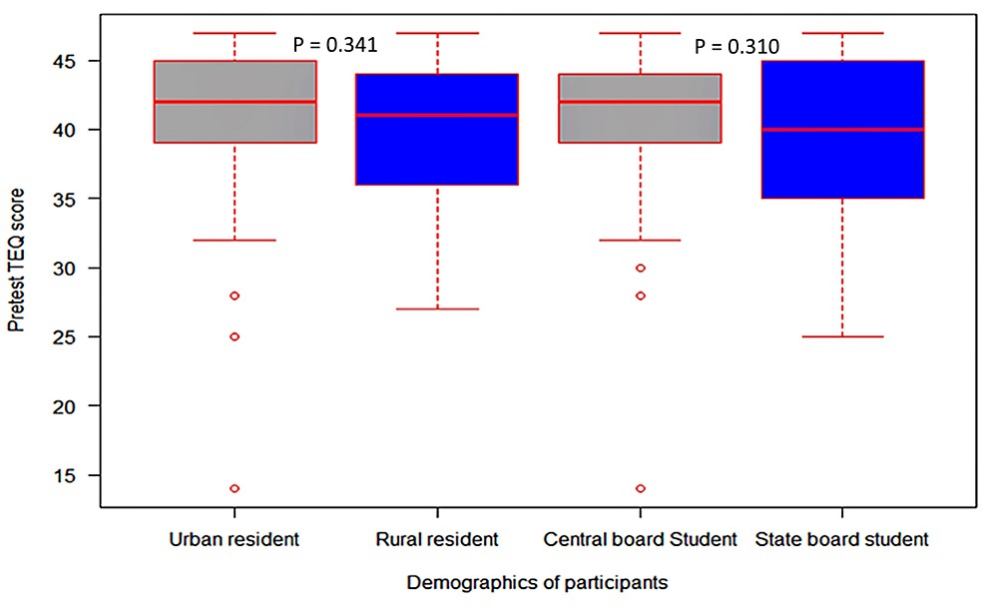
Comparison of pre-test TEQ scores based on the type of schooling and place of residence. TEQ: Toronto Empathy Questionnaire.

**Figure 3:**
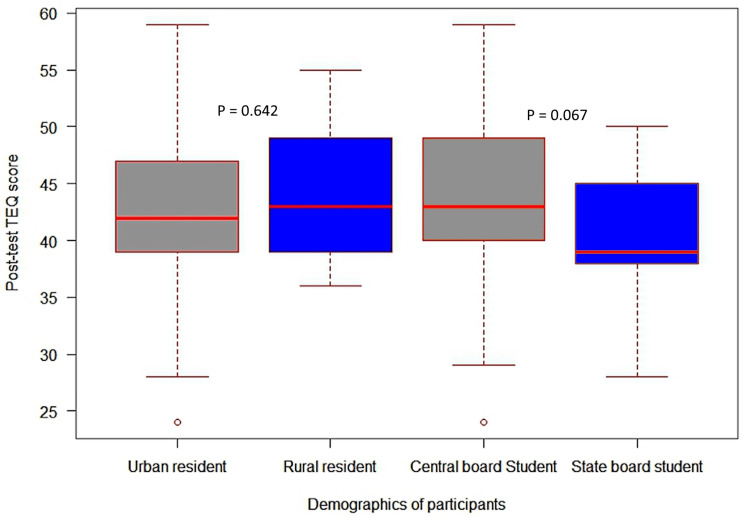
Comparison of post-test TEQ scores based on the type of schooling and place of residence. TEQ: Toronto Empathy Questionnaire.

Figures 2, 3 illustrate the variation in participants' scores based on the type of schooling and place of residence. Participants who lived in urban areas had higher baseline scores than those in rural areas. Similarly, participants who attended central board schools had higher baseline scores than those in state board schools. However, no statistically significant difference (p=0.310) was observed in baseline and post-test TEQ scores based on the type of schooling and place of residence.

**Figure 4:**
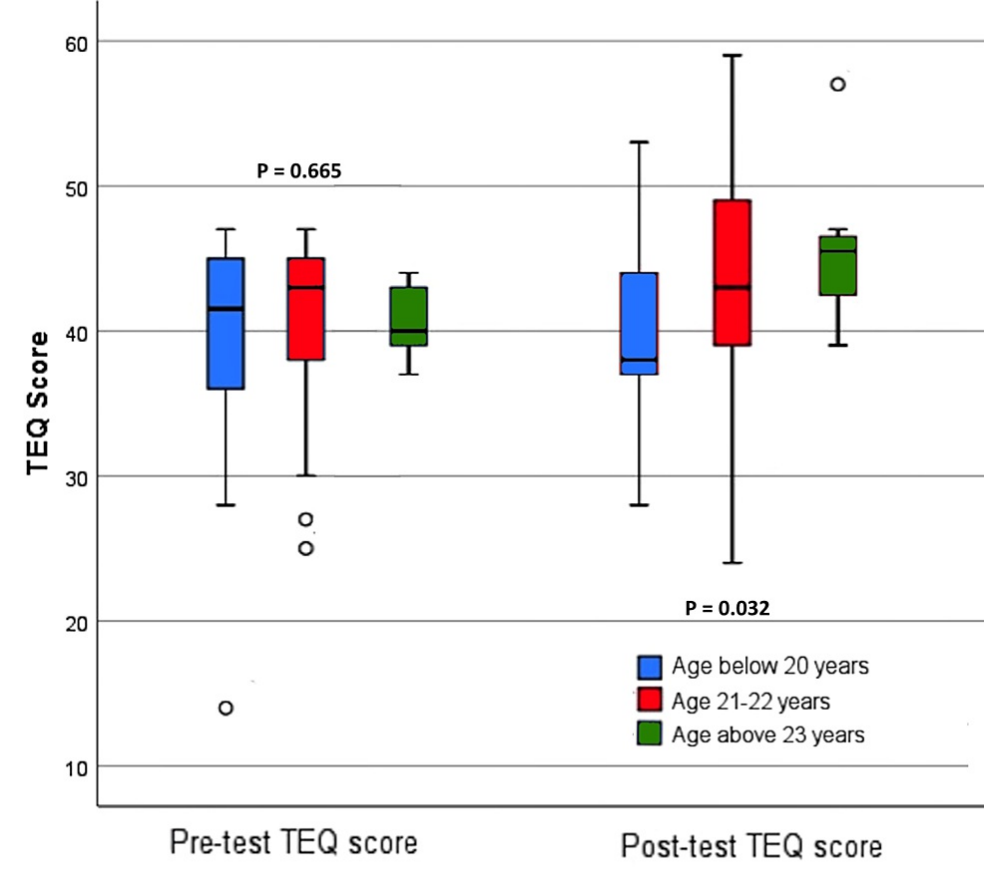
Pre-test and post-test TEQ scores based on the different age categories of the participants. TEQ: Toronto Empathy Questionnaire.

Figure 4 presents the post-test TEQ scores of the participants across different age groups. Participants in the higher age group had higher median scores than those in the lower age group for the pre-test score. However, the difference was not statistically significant (p=0.665). Participants in the higher age group had significantly (p=0.032) higher median post-test scores than those in the lower age group. The association of age with the post-test TEQ score was positive, weak, but significant (Spearman's rho: 0.277, p=0.014), whereas with the pre-test, the association was non-significant (Spearman's rho: 0.095, p=0.404).

Comparison of pre-and post-test subscale and total TEQ scores

Table 2 indicates a statistically significant improvement (p=0.009) in the total TEQ scores following the study intervention. Also, statistically significant (p=0.005) improvement was seen in subgroup III, which assesses emotional states in others by indexing the frequency of behaviors and demonstrating appropriate sensitivity, and subgroup IV (p=0.016), which evaluates sympathetic physiological arousal.

**Table 2:** Subgroups of empathy according to the TEQ. TEQ: Toronto Empathy Questionnaire.

Domains of empathy	Pre-test		Post-test		p (Wilcoxon signed-rank test)
	Mean	SD	Mean	SD	
Perception of emotion in another that stimulates the same emotion in oneself	4.582	1.2670	4.848	1.4596	0.254
Assessment of emotion comprehension in others	2.620	0.9241	2.544	1.0108	0.621
Assessment of emotional states in others by indexing the frequency of behaviors, demonstrating appropriate sensitivity	12.076	2.6398	13.367	3.0474	0.005
Sympathetic physiological arousal	9.671	2.2967	10.595	1.9646	0.016
Altruism	8.734	2.0109	8.835	2.0782	0.946
Behaviors engaging higher-order empathic responding	2.835	0.8977	2.949	0.9323	0.630
Total TEQ score	40.519	5.7062	43.139	6.8064	0.009

Association of demographic characteristics and post-test TEQ scores

The univariate analysis of variance revealed that there was no statistically significant difference in post-test TEQ scores based on gender (Table 3), type of schooling (Table 4), or place of residence (Table 5). The partial eta squared for gender, type of schooling, and place of residence were (ηp^2^=0.017, p=0.255), type of schooling (ηp^2^=0.037, p=0.091), and place of residence (ηp^2^=0.006, p=0.431).

**Table 3:** Univariate analysis of variance for post-test TEQ scores and gender. TEQ: Toronto Empathy Questionnaire. ^a^R^2^=0.018 (adjusted R^2^ = -0.008). Dependent variable: post-test.

Source	Type III sum of squares	df	Mean square	F	Sig.	Partial eta squared
Corrected model	64.046^a^	2	32.023	0.686	0.507	0.018
Intercept	2912.000	1	2912.000	62.352	0.000	0.451
Gender	61.482	1	61.482	1.316	0.255	0.017
Pre-test	15.937	1	15.937	0.341	0.561	0.004
Error	3549.422	76	46.703			
Total	150632.000	79				
Corrected total	3613.468	78				

**Table 4:** Univariate analysis of variance for post-test TEQ scores and type of schooling. TEQ: Toronto Empathy Questionnaire. ^a^R^2^=0.038 (adjusted R^2^ = 0.013). Dependent variable: post-test

Source	Type III sum of squares	df	Mean square	F	Sig.	Partial eta squared
Corrected model	136.927^a^	2	68.463	1.497	0.230	0.038
Intercept	3053.005	1	3053.005	66.741	0.000	0.468
Type of schooling	134.362	1	134.362	2.937	0.091	0.037
Pre-test	8.996	1	8.996	0.197	0.659	0.003
Error	3476.542	76	45.744			
Total	150632.000	79				
Corrected total	3613.468	78				

**Table 5:** Univariate analysis of variance for post-test TEQ scores and place of residence TEQ: Toronto Empathy Questionnaire. ^a^R^2^=0.006 (adjusted R^2^ = -0.020). Dependent variable: post-test.

Source	Type III sum of squares	df	Mean square	F	Sig.	Partial eta squared
Corrected model	22.908^a^	2	11.454	0.242	0.785	0.006
Intercept	2981.702	1	2981.702	63.113	0.000	0.454
Place of residence	20.343	1	20.343	0.431	0.514	0.006
Pre-test	1.790	1	1.790	0.038	0.846	0.000
Error	3590.561	76	47.244			
Total	150632.000	79				
Corrected total	3613.468	78				

## Discussion

The study aimed to evaluate the effect of a multimodal intervention on improving medical students' empathy scores and to explore potential differences in empathy scores based on factors such as gender, place of residence, and type of schooling. The post-intervention TEQ scores demonstrated a statistically significant improvement. Additionally, the study found that factors such as gender, type of schooling, and place of residence did not substantially impact empathy scores, suggesting that the multimodal intervention was effective across different demographics.

The mean baseline TEQ score in our study (40.519±5.71) was comparable to a previous study (39.28±15.65) [14]. However, the mean baseline TEQ score was lower than studies conducted in other countries, such as the Caribbean and Romania, where the average baseline TEQ scores ranged from 45 to 50 [15,16]. This could be attributed to cultural and social differences or variations in the educational systems of these countries [17].

The female participants had higher baseline TEQ scores than the male participants, similar to previous research [18]. One systematic review suggested that, as females are more nurturing and caring by nature, these evolutionary characteristics may account for the difference in baseline empathy scores between male and female participants [19]. However, the post-test TEQ scores for female participants did not show a significant difference from those of the male participants, indicating that the intervention successfully closed the empathy gap that was first noticed between genders. Further, univariate analysis of variance suggests that gender does not significantly impact the post-test score of the participants. The partial eta squared (np^2^=0.032) means that gender has a negligible effect on the post-test score.

Participants in the older age group obtained higher median TEQ values, consistent with earlier research findings [20,21]. Age and post-test TEQ scores also showed a weak but significant positive correlation, implying that older participants experienced more effectiveness from the intervention than younger participants. However, several studies have reported decreased empathy as students progress through their academic years [22-24]. Our findings indicate that age may play a role in increasing empathy levels if targeted interventions are planned and implemented effectively. Further research is needed to explore the factors influencing the relationship between age and empathy levels in medical education.

As per the findings of the univariate analysis of variance, kind of schooling and place of residence do not significantly impact post-test TEQ scores. Also, the partial eta sizes for these factors suggest their negligible effect on post-test TEQ scores. However, higher median scores were reported among students studied in central board schools. Similarly, students from urban areas received higher scores than participants from rural areas. These findings suggest that while the type of schooling and place of residence may not be directly correlated to empathy levels, they may still impact the development and expression of empathy. Our findings contradict a previous study on medical students in India, which found that students from rural backgrounds scored higher on the empathy scale [25]. We could not find any studies that explicitly looked into the relationship between empathy levels and the kind of curriculum (central board or state). More research is required to fully understand the intricate interactions between these factors and the empathy level among students, as there currently needs to be more evidence in this area. 

The significant improvement in the total post-test scores suggests that our multimodal approach helped enhance the participants' empathy levels. Our results are similar to those of studies using mixed-methods interventions such as case studies, discussion in small groups, role-play, and reflection activities [26,27]. One meta-analysis found that interventions involving mixed-methods approaches had the most significant improvement in empathy levels compared to interventions solely relying on one method [28]. Similarly, D'Souza et al. reported that even a single communication skills training session using PowerPoint, video clips, and role-play significantly increased the empathy level among students, which was maintained even after three weeks of training [29].

Sathaporn and Pitanupong suggested that providing experiential learning, opportunities to experience the patient's perspective, and inclusion of early clinical exposure in the curriculum, along with adequate opportunities to reflect on interactions with the patient, can effectively enhance the empathy level among medical students [30]. The multimodal approach used in this research is similar to the above-proposed model. By incorporating role-playing activities using different case scenarios and providing opportunities for students to engage in reflective discussions, we can further enhance their ability to comprehend patients' feelings.

The study's strength is implementing a multimodal interventional design, in contrast to most prior non-interventional cross-sectional studies done on medical students in India. This holistic approach adds credibility and richness to our findings, making them more reliable and informative for future research and interventions. Additionally, the study's focus on second-year medical students allows for developing targeted interventions early in their medical education to improve and maintain their empathy levels during the latter part of their academic career.

Limitations

Despite the strengths mentioned above, there are a few limitations to consider. This study was conducted in a single center among second-year medical students, so the findings may not be generalized to other regions or students in different years of medical school. A self-report measure assessed empathy levels, which may be subject to bias and social desirability. Future research could overcome these limitations by including a more extensive and diverse sample and utilizing objective measures of empathy. Follow-up measurements were not conducted to assess any changes in empathy levels over time, limiting our understanding of the long-term effects of intervention on empathy development. Future studies could address this by implementing longitudinal designs and tracking empathy levels periodically. Exploring other factors that may influence empathy, such as stress level and other psychological factors, would be beneficial to gain a more comprehensive understanding of the factors contributing to empathy development in medical students.

## Conclusions

The multimodal intervention resulted in a significant improvement in the TEQ scores of medical students. The results also emphasize how focused interventions can lessen the disparities in empathy levels between genders. The findings of this study have important implications for medical education, highlighting the effectiveness of incorporating various methods to enhance empathy in future healthcare professionals. Furthermore, the study found no significant differences in empathy scores based on factors such as gender, place of residence, or type of schooling, indicating that the proposed multimodal intervention's benefits may apply to all medical students regardless of their background.
